# High expression of CCDC69 is correlated with immunotherapy response and protective effects on breast cancer

**DOI:** 10.1186/s12885-023-11411-2

**Published:** 2023-10-12

**Authors:** Zhen Wang, Huiyang Ren, Guolian Zhu, Lei Zhang, Hongyi Cao, Bo Chen

**Affiliations:** 1 Department of Breast and Thyroid Surgery, Shandong Provincial Hospital Affiliated to Shandong First Medical University, Jinan, China; 2 Department of Breast Surgery, The First Hospital of China Medical University, Shenyang, China; 3 Department of Breast Surgery, The Fifth People’s Hospital of Shenyang, Shenyang, China; 4 Department of Pathology, The First Hospital of China Medical University and College of Basic Medical Sciences, Shenyang, China

**Keywords:** CCDC69, Immune infiltration, Immunotherapy, Breast cancer, Biomarker

## Abstract

**Background:**

As a molecule controlling the assembly of central spindles and recruitment of midzone component, coiled-coil domain-containing protein 69 (CCDC69) plays an important role in multiple cancers. Currently, the relationships between CCDC69 and immune infiltration or immunotherapy in breast cancer remain unclear.

**Methods:**

The expression and prognostic significance of CCDC69 in breast cancer were comprehensively analyzed by quantitative real-time PCR, immunohistochemical staining and various databases. The data source of differentially expressed genes, gene set enrichment analysis, and immune cell infiltration analysis came from The Cancer Genome Atlas (TCGA) database. Single-cell analysis based on IMMUcan database was used. The protein-protein interaction network was developed applying STRING, Cytoscape, CytoHubba, and GeneMANIA. TISIDB was employed in analyzing the CCDC69 co-expressed immune related genes. The correlations between CCDC69 and immunotherapy or immune-related scores were analyzed by CAMOIP and TISMO. Ctr-db was also used to conduct drug sensitivity analysis.

**Results:**

The mRNA of CCDC69 was downregulated in breast cancer tissues compared with normal tissues. Higher CCDC69 expression was associated with a better breast cancer prognosis. Enrichment analysis showed that the co-expression genes of CCDC69 were mainly related to immune-related pathways. The expression of CCDC69 was found to be positively correlated with multiple tumor-suppression immune infiltration cells, especially T cells and dendritic cells. Meanwhile, high CCDC69 expression can predict better immunotherapy responses when compared with low CCDC69 expression. After the interferon-gamma treatment, the CCDC69 expression was elevated in vitro. CCDC69 expression was a reliable predictor for the response status of two therapeutic strategies in breast cancer.

**Conclusions:**

Our research revealed the clinical significance of CCDC69 in breast cancer and validated the critical roles of CCDC69 in the tumor immune infiltration and immunotherapy responses.

**Supplementary Information:**

The online version contains supplementary material available at 10.1186/s12885-023-11411-2.

## Introduction

 Study showed that breast cancer is the most frequently diagnosed cancer in 2022 in the USA [1] and the fifth leading cause of cancer mortality worldwide [2]. The overall 5-year survival rate for breast cancer patients with metastasis is only 23% [3]. Breast cancer is highly heterogeneous, and its progression is a complex process that can be influenced by microenvironment and patients’ immune system [4, 5]. Immune system cells participate in various life activities and exert effects on the clinical outcomes of cancers [6]. Growing evidence indicated that high level of immune infiltration is correlated with better survival and response to treatment, especially for immunotherapy in breast cancer [4, 7–9].

Coi led-coil domain-containing protein 69 (CCDC69), which locates on 5q33.1, has been demonstrated to play a critical role in controlling the assembly of central spindles and recruitment of midzone component. Recent studies showed that CCDC69 also functions in ovarian cancer [10], colon cancer [11], gastric cancer [12], breast cancer [13], and lung cancer [14]. Wang et al. considered CCDC69 as a hub gene related to the immune microenvironment in colon cancer [11]. Cui et al. revealed that CCDC69 could enhance platinum-induced apoptosis in ovarian cancer [10], and they further verified that the overexpression of CCDC69 could activate p14ARF/MDM2/p53 pathway and confer cisplatin sensitivity [15]. Also, CCDC69 has also been reported to be significantly related to the survival of breast patients [13]. A machine learning study based on TCGA database showed that CCDC69 expression is negatively correlated with tumor purity [16]. These findings all suggested the prognostic and underlying therapeutic value of CCDC69 in cancers. Currently, comprehensive study of CCDC69 in breast cancer has not been conducted. Moreover, the relationships between CCDC69 and immune infiltration and immunotherapy response in breast cancer remains unclear.

This paper first analyzed the expression and prognostic value of CCDC69 in using clinical breast cancer samples from patients and multiple bioinformatics databases. Protein-protein interaction (PPI) networks were produced. Gene set enrichment analysis (GSEA) was also performed. This study demonstrated the associations of CCDC69 with clinical features, immune infiltration, and immunotherapy in breast cancer. In conclusion, the upregulation of CCDC69 was correlated with favorable prognosis and immunotherapy benefits for breast cancer patients.

## Methods and materials

### Patients and samples

Breast cancer and adjacent normal tissues were collected after surgery from the First Hospital of China Medical University and it was approved by Ethics Committee of the First Hospital of China Medical University (Number: AF-SOP-07-1.1-01). All the patients were diagnosed clearly by pathologists. Patients diagnosed with other malignant tumors were excluded. We finally collected 36 pairs of tumor and adjacent normal tissues for quantitative reverse transcriptase-polymerase chain reaction (qRT-PCR) for differential expression verification. Besides, 101 tumor tissues with follow-up data collected were used for survival analysis.

### RNA extraction and qPT-PCR

TRIzol reagent (Invitrogen, USA) was used for the extraction of total RNA. The purity and concentration of the RNA extracts were successively verified by spectrophotometry (A260/A280 ratio should be between 1.8 and 2.0). The Vazyme HIScript II RT SuperMix for qPCR (+ Gdna wiper) Kit was used for the synthesis of cDNA, and Vazyme SYBR Green qPCRmix was used for qPT-PCR. The 2-ΔΔCt method was applied to analyze the relative expression level which was normalized to GAPDH expression. The primers are shown below:

CCDC69 forward: 5′−CTGTCCAGCTCTGTGCATCAGA − 3′,

CCDC69 reverse: 5′−CTGCTCATCCAGTCTGTCTCGA − 3′.

GAPDH forward: 5′−GGAGCGAGATCCCTCCAAAAT − 3′,

GAPDH reverse: 5′−GCTGTTGTCATACTTCTCATGGG − 3′.

### Immunohistochemistry

After dehydration and paraffin-embedding, the breast tissues were fixed with 4% paraformaldehyde and prepared as tissue sections. After dewaxing and hydration, we used Citrate buffer for antigen retrieval at 95℃ for 15 min (min). Next, after cooling to room temperature, 3% H_2_O_2_ was used to block the endogenous peroxidase activity. Then, the sections were incubated with primary antibody CCDC69 (Novus, NBPI-85,139, 1:200) overnight at 4℃. After that, secondary antibodies incubation, DAB regents (Maxim, DAB-0031/1031) staining, and hematoxylin counterstaining were performed. Two pathologists were invited to evaluate the immunohistochemical results of each section. When disagreement about the results arouse, a third pathologist was invited to independently evaluate the results. After excluding nonspecific staining, cells with clear brown-yellow granulosa in the nucleus or cytoplasm area were defined as positive cells under a microscope.

### Assessment of CCDC69 differential expression on clinical samples and bioinformatics platforms

Gene Expression patterns across Normal and Tumor tissues database (GENT2) (http://gent2.appex.kr/gent2/) is an updated version of GENT providing a user-friendly search of gene expression patterns across different normal and tumor tissues compiled from public gene expression data sets. The current pan-cancer expression analysis was conducted based on GENT2. RNA-seq data of BRCA in level 3 HTSeq-FPKM were downloaded from official TCGA website and further transformed into transcripts per million reads (TPM) format. The expression data based on TCGA database and qPT-PCR outcomes were analyzed by R (version 3.6.3) and R package ggplot2(version 3.3.3) and Graphpad prism(version 8.0.2).

### Assessment of the prognosis value of CCDC69 on survival

The Kaplan-Meier Plotter platform (www.kmplot.com) is an online database including gene expression data and clinical data. With the purpose to assess prognostic value of a specific gene, the platform was applied in drawing the Kaplan-Meier (KM) survival curves for patients with different CCDC69 expression levels [17]. The R package “survminer (version 0.4.9)” and “survival (version 3.2–10)” was used to analyze patients’ survival data in TCGA database and clinical follow-up dataset of IHC staining group. In the Cox univariate and multivariate regression analysis, factors with a p value more than 0.1 in the univariate analysis were enrolled in the multivariate analysis. R package “survival (version 3.2–10)” was also used in this section.

### Identification of differentially expressed genes

R package “DESeq2 (version 1.26.0)” was used to filter differentially expressed genes (DEGs) [18] (p.adj < 0.05, |log2FoldChange|>2) between high expression group and low expression group of CCDC69 divided by the median value in TCGA database. The R package “ggplot2 (version 3.3.3)” was used to plot the volcano figure.

### Protein–protein interaction network

We used STRING (https://string-db.org) [19] to examine the interactions (required score (median confidence) > 0.4, FDR stringency (medium) > 5%) among the proteins from DEGs. And we applied Cytoscape and CytoHubba (version 0.1) [20] to develop a PPI network and identify the top 15 hub genes. GeneMANIA (https://genemania.org/), which is a flexible user-friendly web site for generating hypotheses about gene function, analyzing gene lists and prioritizing genes for functional assays, was further applied to predict the functions and mechanisms of the selected hub genes [21].

### GSEA of all the detected genes

GSEA software (version 4.0.3) [22] was used to conduct GSEA for identifying potential enriched functions and pathways of CCDC69-correlated gene set. The c5.all.v7.0.symbols.gmt data sets were downloaded from the MsigDB database (http://www.broad.mit.edu/gsea/msigdb/index.jsp) on the GSEA website. The default weighted enrichment statistics method was used, and the number of random combinations was set to 1000 times.

### Analysis in breast cancer gene-expression miner (bc-GenExMiner) v4.8

The correlations between CCDC69 and ER status, PR status, HER2 status, nodal status, histological types, and PAM50 subtypes were explored using bc-GenExMiner v4.8, which is a statistical mining tool for published breast cancer transcriptomic data [23].

### Immune infiltration analysis

The enrichment score was defined by the single sample GSEA to represent the absolute enrichment degree of a gene set in each sample within a given dataset using R package “GSVA” [24]. We also calculated the normalized enrichment scores for each immune category. Various immune cell gene set signatures were obtained from a previous study [25]. We further evaluated the associations between CCDC69 expression and immunomodulators and chemokines in Tumor-Immune System Interactions database (TISIDB) (http://cis.hku.hk/TISIDB), which is an online integrated repository portal containing abundant human cancer datasets from the TCGA database [26].

### Single cell analysis

We downloaded BC_UNB_10X_E - MTAB – 8107 and TNBC_IMM_10X_GSE169246 breast cancer datasets in h5ad format from IMMUcan database (https://immucanscdb.vital-it.ch/). And the data were further transferred into rds format by sceasy package. In the follow-up analysis, R package Seurat (version: 4.2.0) was adopted for follow-up analysis. The entire analysis was performed in the R environment.

###  Immunotherapy response and immune-related score analysis

We detected the expression level of CCDC69 in mouse samples in vivo from immune checkpoint inhibitor (ICI) studies as well as in vitro samples with cytokines treatment from Tumor Immune Syngeneic Mouse database (TISMO) [27], which is a database for investigating and visualizing gene expression, pathway enrichment, and immune cell infiltration levels in syngeneic mouse models across different immune checkpoint blockade (ICB) treatment and response groups in 23 cancer types. The survival curve and box plots were generated from CAMOIP database, a web server for comprehensive analysis on multi-omics of immunotherapy in pan-cancer (https://www.camoip.net/).

### Drug sensitivity analysis

We explored the predictive value of CCDC69 under different therapeutic strategies for treating breast cancer by Cancer Treatment Response gene signature DataBase (ctr-db) (http://ctrdb.cloudna.cn/) [28]. CTR_Microarray_92 and CTR_Microarray_74 were analyzed. The ability to predict drug response was based on the AUC value.

### Data presentation and statistical analysis

The quantitative data downloaded from various bioinformatics platforms were shown as the mean plus the standard error of the mean. Shapiro-Wilk normality test, Levene’s test, paired and unpaired samples t test, and Wilcoxon signed rank test were performed to compare the expression between the two groups. For two independent samples, we first used Shapiro-Wilk normality test and Levene’s test to assess the normality and homogeneity of variance, and if they all met the criteria, unpaired t test was applied, otherwise Wilcoxon signed rank test was used. For paired samples, Shapiro-Wilk normality test was first used to test the normality, and if the samples were normally distributed, paired t test was used, otherwise Wilcoxon signed rank test was used. Spearman correlation test was performed to evaluate the correlations in the immune infiltration analysis. In the survival analysis, univariate and multivariate Cox regression models were employed to investigate the relationship between clinical factors and survival. Survival curves were compared by log-rank test. And the *p* < 0.05 was considered as statistically significant. And Graphpad Prism 8.0.2 was used to visualize the qPT-PCR results and the data downloaded from TISMO database. All the other statistical analyses were performed using R software (version 3.6.3).

## Results

### CCDC69 was low-expressed in cancers

**Fig. 1:**
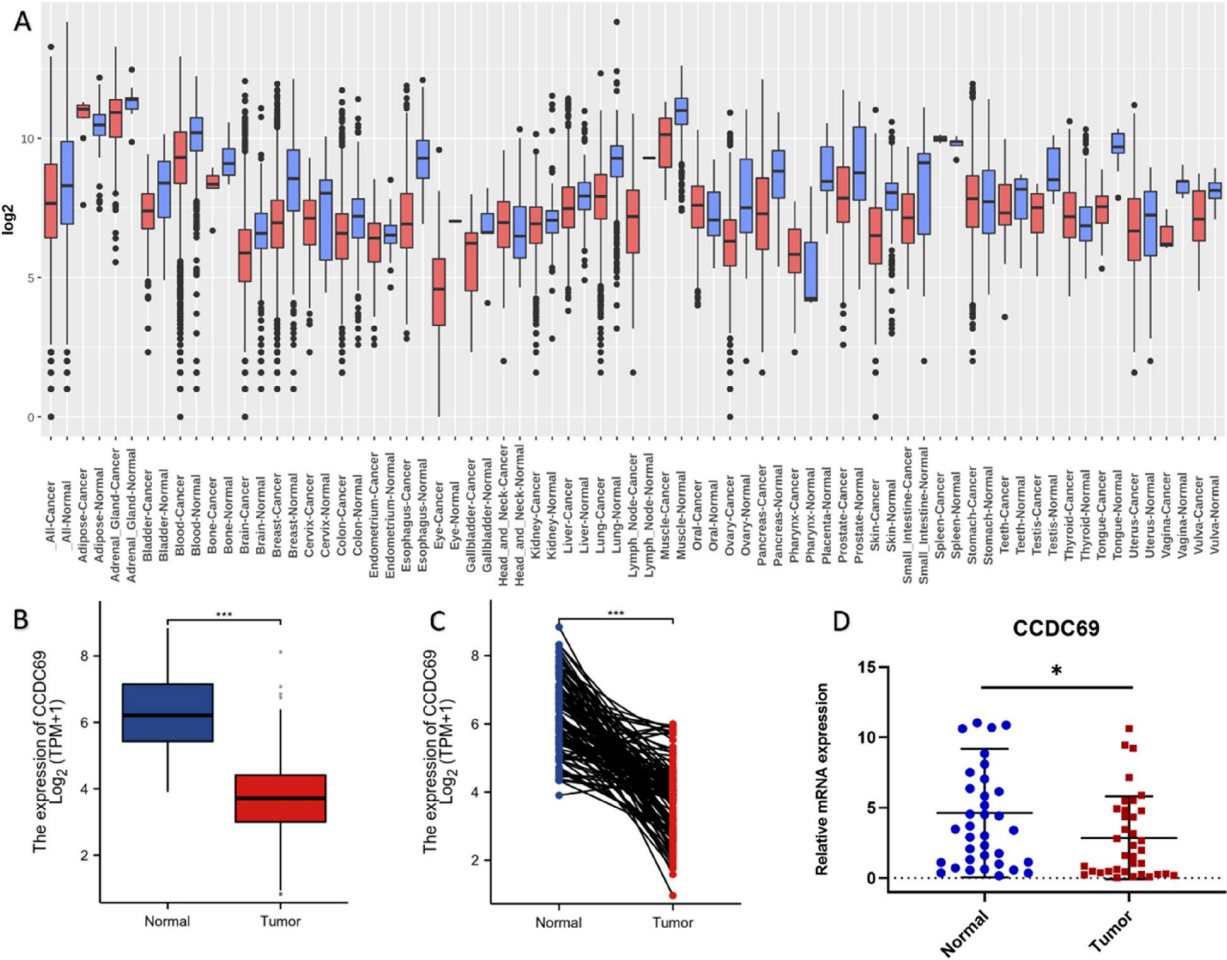
CCDC69 was downregulated in human breast cancer. **A** Transcription levels of CCDC69 in different types of cancer and normal tissues using GENT2 database. **B** Relative mRNA expression of CCDC69 in the unpaired breast cancer and normal tissues in TCGA database. **C** Relative mRNA expression of CCDC69 in the paired breast cancer and normal tissues in TCGA database. **D** qRT-PCR of CCDC69 expression in 36 human breast cancer tissues and their paired adjacent nontumor tissues. ***p < 0.001, **p < 0.01, *p < 0.05, ns: not significant

Figure 1 A showed the gene expression of CCDC69 in 33 different types of human cancers. We found that CCDC69 was differently expressed in 18 cancer types with statistical significance in lung, blood, brain, breast, skin, colon, ovary, pancreas, esophagus, tongue, adrenal gland, prostate, kidney, bladder, liver, vulva, vagina, and endometrium cancers. CCDC69 was downregulated in the 18 types of cancer tissues compared with adjacent normal tissues. The p values and log_2_FoldChange can be found in Supplementary Fig. 1. We further verified that CCDC69 was lower-expressed in breast cancer tissue compared with adjacent normal tissue both in TGCA database ( independent samples : unpaired t test, *p*<0.001; paired samples: paired t test, *p*<0.001; Fig. 1B,C) and in patients’ samples using qRT-PCR (Wilcoxon signed rank test, *p* = 0.0168; Fig. 1D).

### High expression of CCDC69 predicts a favorable prognosis in breast cancer

**Fig. 2:**
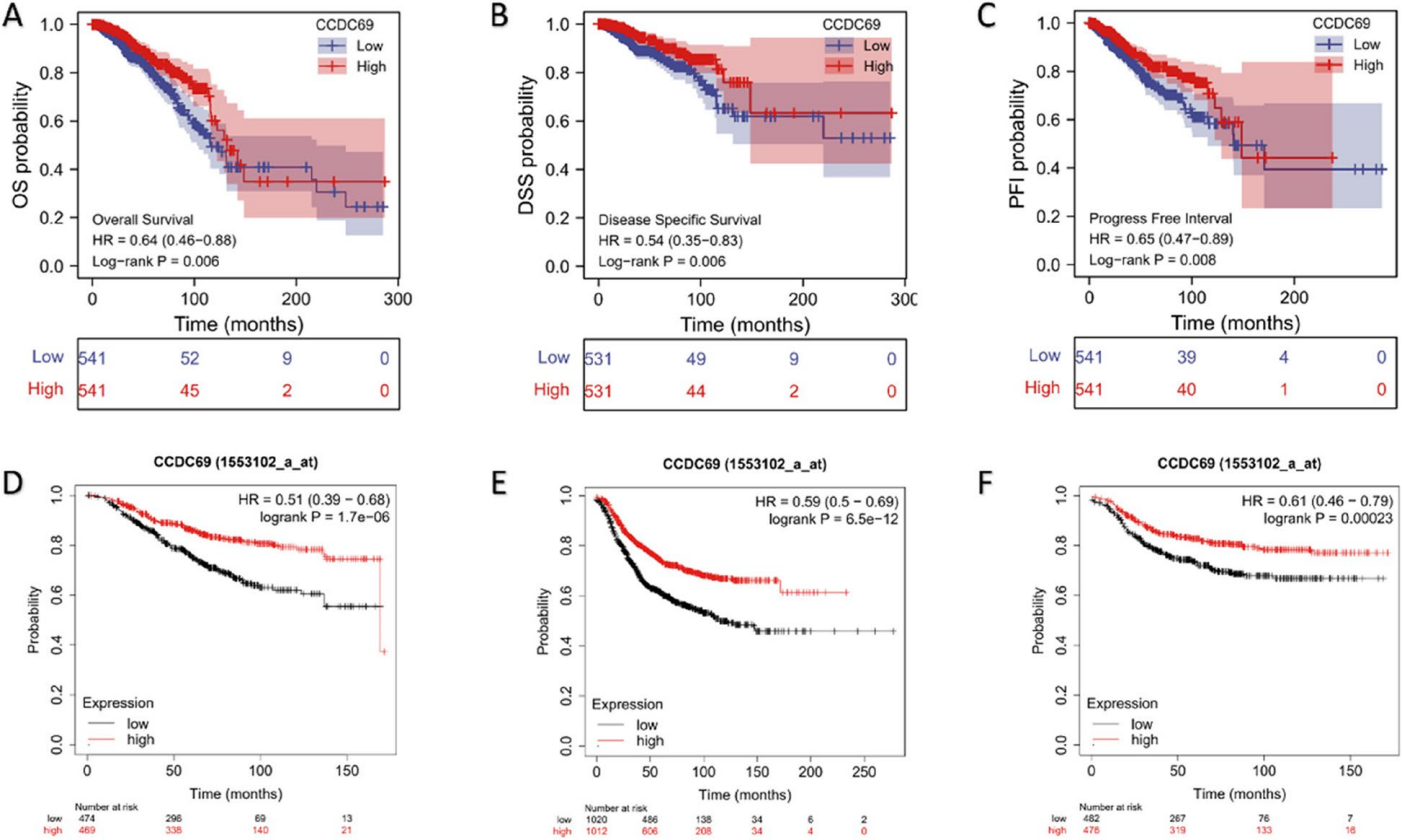
CCDC69 upregulation was correlated with longer survival in patients with breast cancer. The KM survival curve of OS (**A**), DSS (**B**), and PFI (**C**) of breast cancer patients divided by CCDC69 expression in TCGA database, and OS (**D**), RFS (**E**), and DMFS (**F**) of breast cancer patients divided by CCDC69 expression in Kaplan-Meier platform

**Fig. 3:**
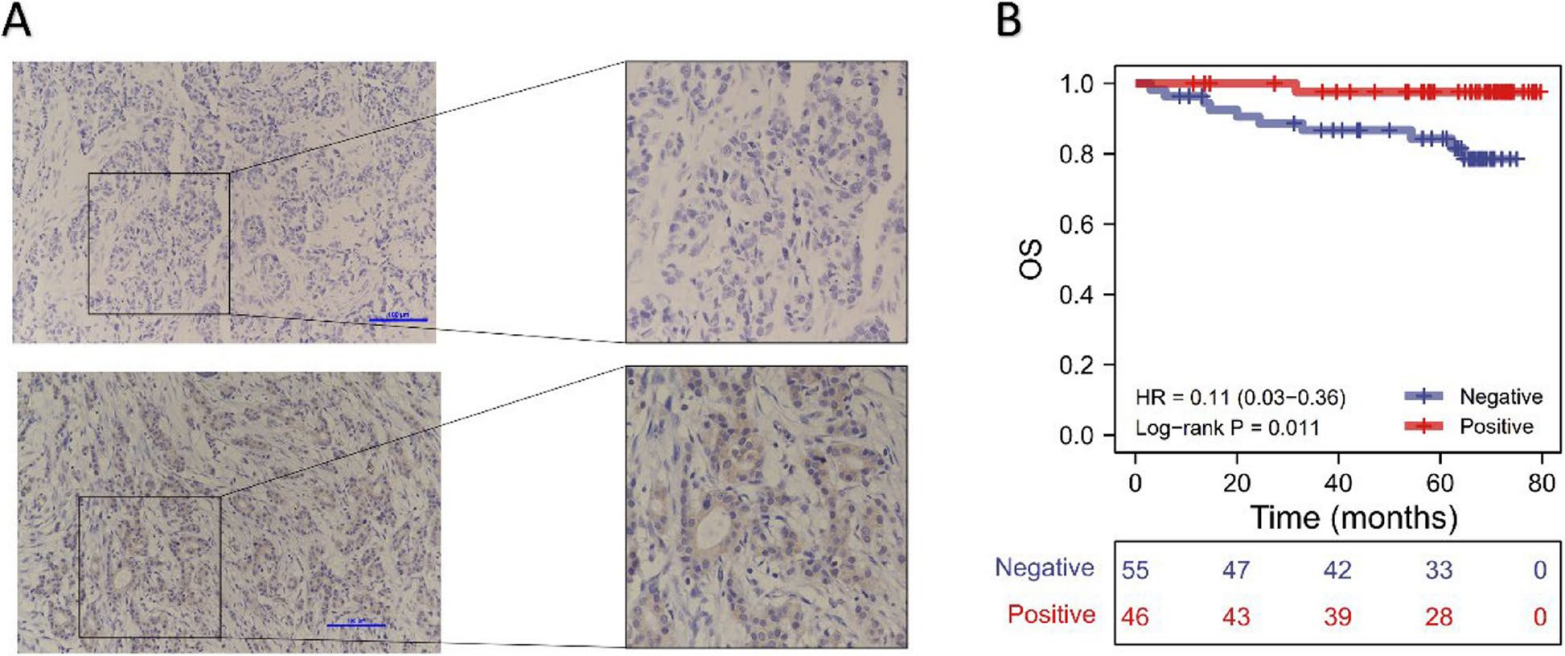
**A** Representative IHC staining of CCDC69. **B** The KM survival curve of OS of 101 breast cancer patients whose CCDC69 expression was evaluated by IHC staining

The above analysis indicated that CCDC69 expression was significantly downregulated in breast cancer. To explore the prognostic value of CCDC69, we first plotted the KM survival curve using TCGA database and Kaplan-Meier Plotter platform. Patients were divided into low expression group and high expression group according to the median value of CCDC69 expression. In the overall survival (OS) analysis (Fig. 2A and D), disease-specific survival (DSS) analysis (Fig. 2B), progression-free interval (PFI) analysis (Fig. 2C), recurrence-free survival (RFS) analysis (Fig. 2E), and distant metastasis-free survival (DMFS) analysis (Fig. 2F), high expression of CCDC69 was always a protective factor in breast cancer with statistical significance. We further applied IHC staining to detect CCDC69 expression in a total of 101 breast cancer samples with clinical follow-up data, and found that the median follow-up time was 64.87 months (Fig. 3A). KM survival analysis (Fig. 3B) was then performed. And the outcomes indicated that the CCDC69-positive group (*n* = 46) had longer OS time (hazard ratio (HR) = 0.11, 95% confidence interval (CI) (0.03–0.36), *p* = 0.011) compared with CCDC69-negative group (*n* = 55).

**Fig. 4:**
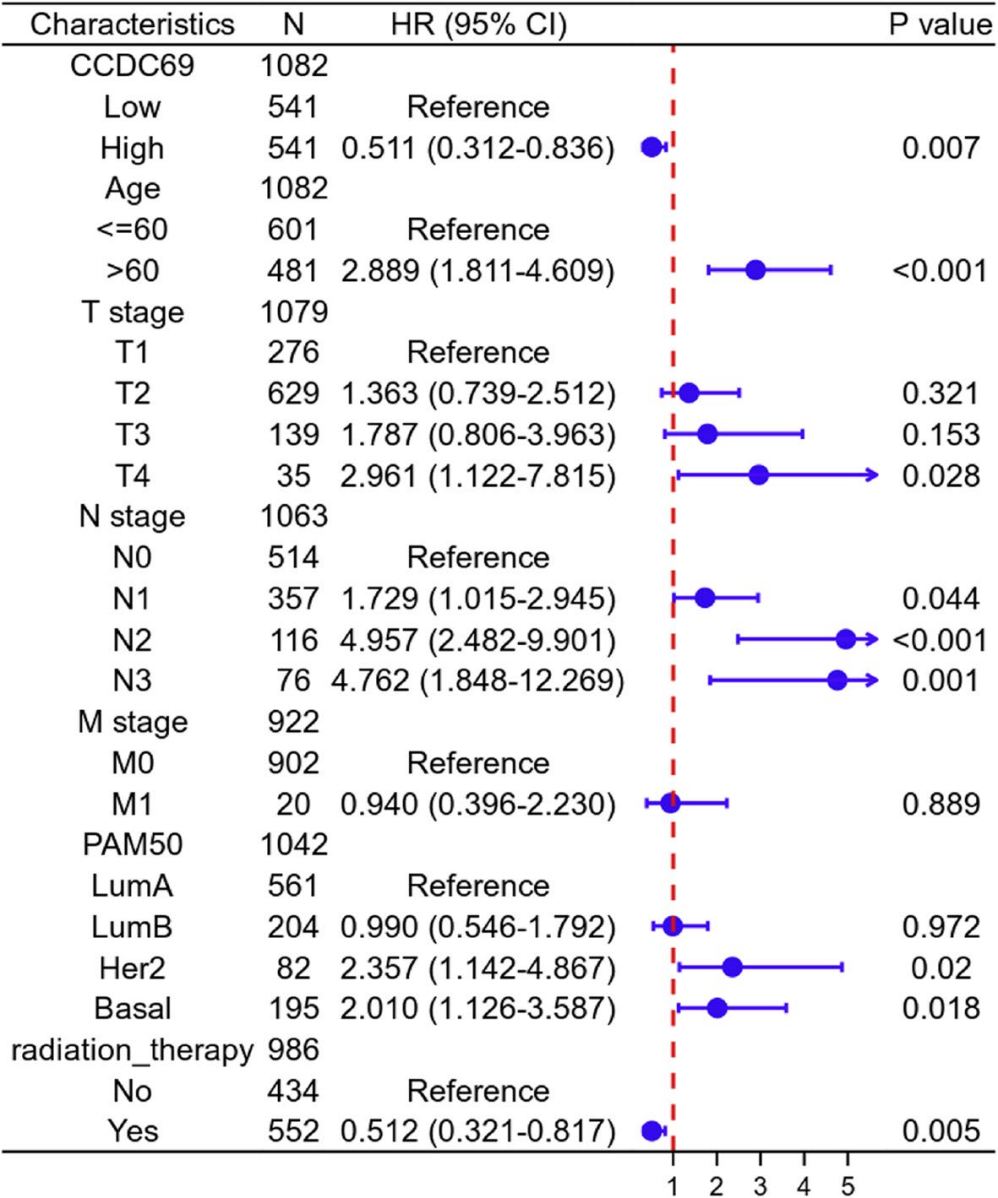
Forest map of multivariate cox analysis of the relationship between CCDC69 expression and OS of TCGA breast cancer patients. ***p < 0.001, **p < 0.01, *p < 0.05, ns: not significant

Using the Cox regression model, we computed both univariate and multivariate hazard ratios for different variables of 1082 breast cancer patients in TCGA database. Univariate Cox regression analysis (Table 1) demonstrated that CCDC69 expression level was an independent variable (high versus low, HR = 0.635 95%CI (0.458–0.881), *p* = 0.007) to predict the OS of breast cancer patients. Multiple Cox regression analysis (Table 1; Fig. 4) also revealed that CCDC69 expression level was an independent factor (high versus low, HR = 0.511, 95%CI (0.312–0.836), *p* = 0.007) of the OS of patients with breast cancer after adjustment for age, TNM stage, PAM50 classification, and radiation therapy status. Similar results were observed in the univariate and multivariate Cox regression analysis on DSS (Supplementary Tables 1 and Supplementary Fig. 2) and PFI (Supplementary Tables 2 and Supplementary Fig. 3). These results also confirmed that the expression level of CCDC69 was an independent variable to predict the DSS and PFI of breast cancer patients. All these findings pointed to a favorable prognostic value of CCDC69 in breast cancer.

**Table 1:** Univariate and multivariate cox analysis of the relationship between CCDC69 expression and OS of TCGA breast cancer patients

Characteristics	Total(N)	Univariate analysis		Multivariate analysis	
		Hazard ratio (95% CI)	*P* value	Hazard ratio (95% CI)	*P* value
CCDC69	1082				
Low	541	Reference			
High	541	0.635 (0.458–0.881)	**0.007**	0.511 (0.312–0.836)	**0.007**
Age	1082				
<=60	601	Reference			
> 60	481	2.020 (1.465–2.784)	**< 0.001**	2.889 (1.811–4.609)	**< 0.001**
Race	993				
Asian	60	Reference			
Black or African American	180	1.525 (0.463–5.024)	0.488		
White	753	1.325 (0.420–4.186)	0.631		
T stage	1079				
T1	276	Reference			
T2	629	1.334 (0.889–2.002)	0.164	1.363 (0.739–2.512)	0.321
T3	139	1.572 (0.933–2.649)	0.089	1.787 (0.806–3.963)	0.153
T4	35	3.755 (1.957–7.205)	**< 0.001**	2.961 (1.122–7.815)	**0.028**
N stage	1063				
N0	514	Reference			
N1	357	1.956 (1.329–2.879)	**< 0.001**	1.729 (1.015–2.945)	**0.044**
N2	116	2.519 (1.482–4.281)	**< 0.001**	4.957 (2.482–9.901)	**< 0.001**
N3	76	4.188 (2.316–7.574)	**< 0.001**	4.762 (1.848–12.269)	**0.001**
M stage	922				
M0	902	Reference			
M1	20	4.254 (2.468–7.334)	**< 0.001**	0.940 (0.396–2.230)	0.889
PAM50	1042				
LumA	561	Reference			
LumB	204	1.663 (1.088–2.541)	**0.019**	0.990 (0.546–1.792)	0.972
Her2	82	2.261 (1.325–3.859)	**0.003**	2.357 (1.142–4.867)	**0.020**
Basal	195	1.285 (0.833–1.981)	0.257	2.010 (1.126–3.587)	**0.018**
Radiation therapy	986				
No	434	Reference			
Yes	552	0.576 (0.394–0.841)	**0.004**	0.512 (0.321–0.817)	**0.005**

### PPI network analysis and screening of hub genes

**Fig. 5:**
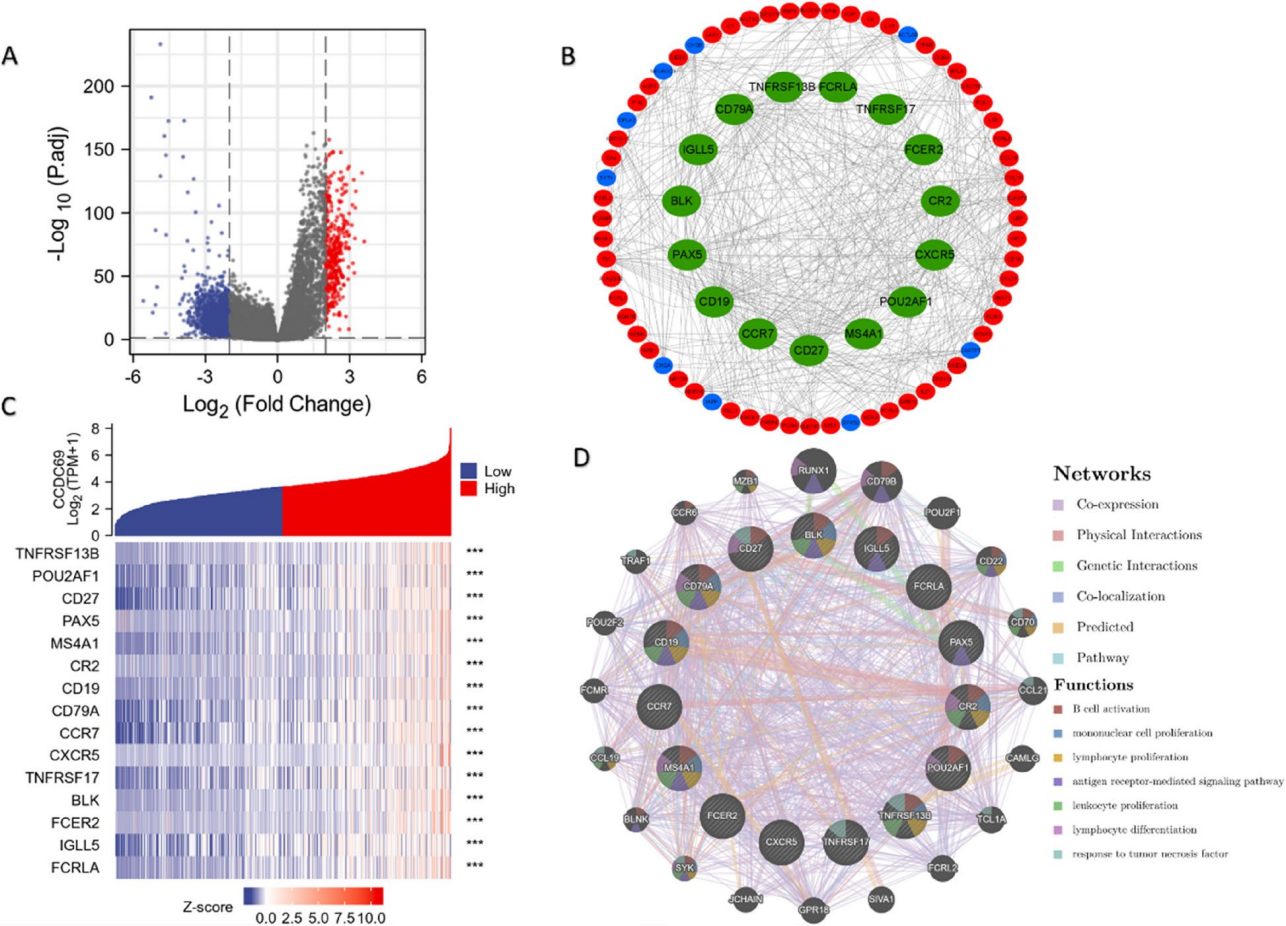
**A** Volcano maps of DEGs from TCGA. **B** The PPI network of the top 15 hub genes created by STRING and Cytoscape. **C** Co-expression heatmap of the 15 hub genes. **D** PPI network and function analyses of the 15 hub genes. Inner circles represent the input genes and outer circles correspond to GeneMANIA proposed hub genes, and the size of the circles indicates the correlation with the input genes

We obtained 1444 DEGs (313 upregulated and 1131 downregulated), and the results were visualized using a volcano plot (Fig. 5A). We identified the top 15 hub genes with the highest interaction scores, and all of them were found to be upregulated (Fig. 5B). The co-expression heat map was shown in (Fig. 5C). These genes were used for the PPI network development with the co-expression of 61.26%, physical interactions of 22.17%, genetic interactions of 5.95%, co-localization of 4.61%, predicted of 4.55%, pathway of 1.46% (Fig. 5D). B cell activation, mononuclear cell proliferation, lymphocyte proliferation, antigen receptor-mediated signaling pathway, leukocyte proliferation, lymphocyte differentiation, and response to tumor necrosis factor were the main functions of those genes.

### GSEA analysis of CCDC69

**Fig. 6:**
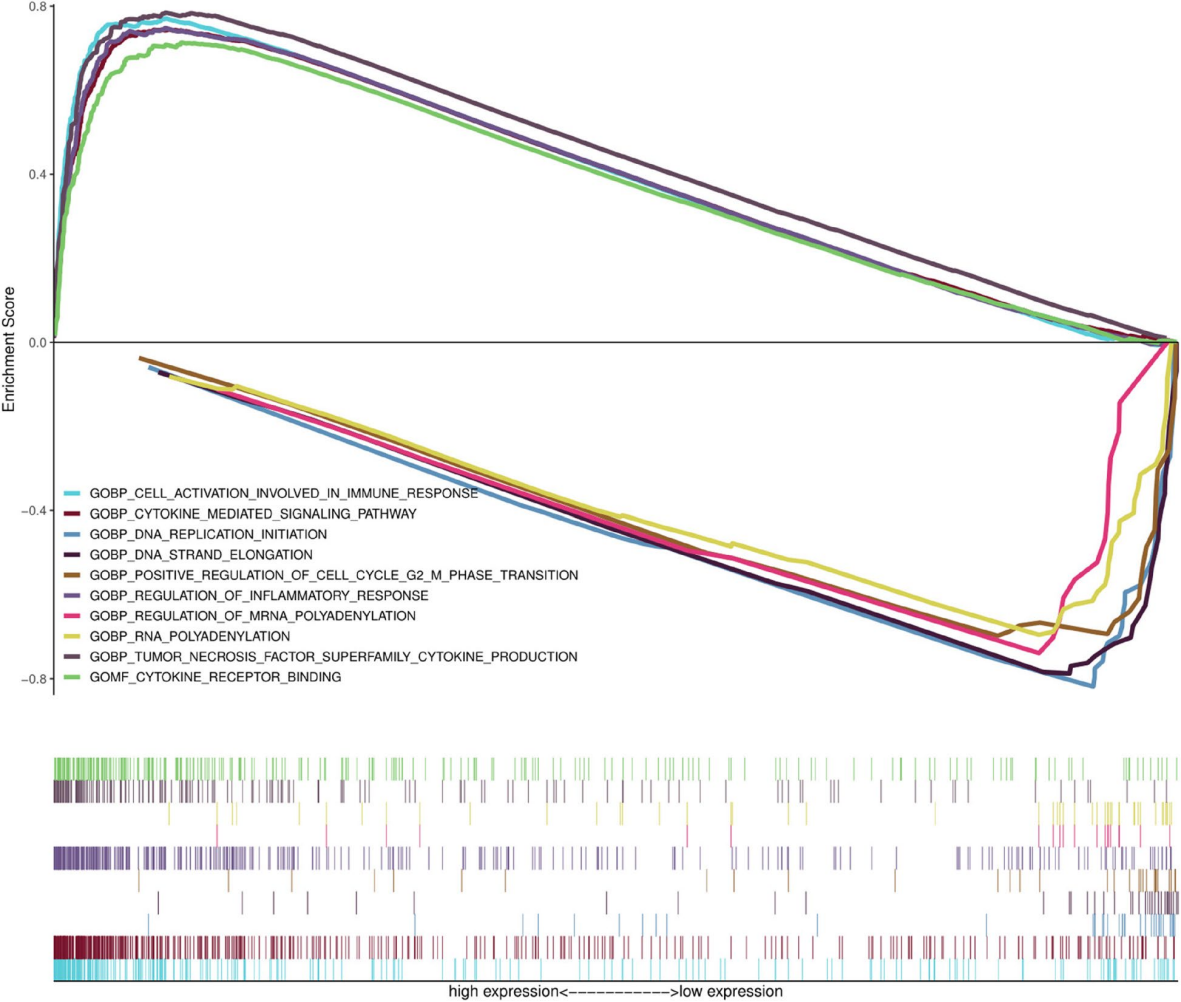
GSEA of GO term analysis revealed five positively correlated groups and five negatively correlated groups

GO analyses were conducted to analyze the potential biological functions and mechanisms of CCDC69. We selected highly enriched signaling pathways based on their normalized enrichment scores. As shown in Fig. 6, GO annotation revealed five categories positively correlated with high levels of CCDC69, namely, cytokine mediated signaling pathway, cytokine receptor binding, tumor necrosis factor superfamily cytokine production, regulation of inflammatory response, and cell activation involved in immune response. GO analysis also uncovered five negatively correlated categories, namely, RNA polyadenylation, DNA strand elongation, DNA replication initiation, regulation of mRNA polyadenylation and positive regulation of cell cycle G2/M phase transition.

### CCDC69 expression is correlated with clinical characteristics in breast cancer

**Fig. 7:**
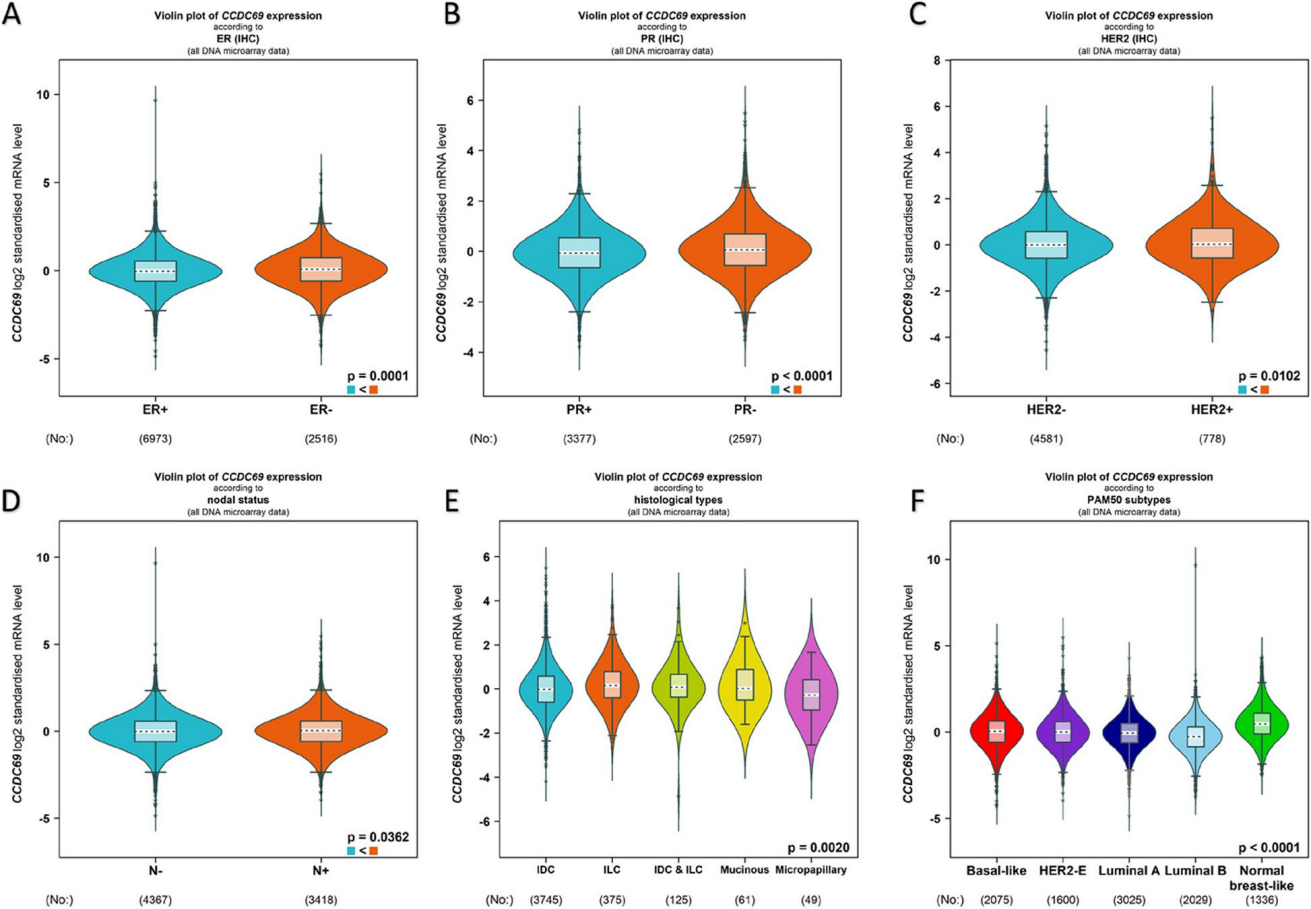
The correlation between CCDC69 expression and the clinical characteristics of breast cancer patients. **A** ER status, (**B**) PR status, (**C**) HER-2 status, (**D**) nodal status, (**E**) histological types, (**F**) PAM50 subtypes

As shown in Fig. 7A-D, CCDC69 was high-expressed in estrogen receptor (ER) -, progesterone receptor (PR) -, human epidermal growth factor receptor 2 (HER2) +, and nodal + groups. Figure 7E indicated that the CCDC69 expression in invasive lobular breast cancer was higher than invasive ductal breast cancer, and that the expression in micropapillary was lower compared with the two groups. Figure 7F showed the comparison of CCDC69 expression among all the PAM50 subtypes (Fig. 7F, pairwise comparison: luminal B < basal like (*p* < 0.0001), luminal B < HER-2-E (*p* < 0.0001), luminal B < luminal A (*p* < 0.0001), normal breast like > basal like (*p* < 0.0001), normal breast like > HER-2-E (*p* < 0.0001), normal breast like > luminal A (*p* < 0.0001), normal breast like > luminal B (*p* < 0.0001), luminal A < basal like (*p* < 0.01), and luminal A < HER-2-E (*p* < 0.01)).

#### CCDC69 is correlated with immune infiltration in breast cancer

**Fig. 8:**
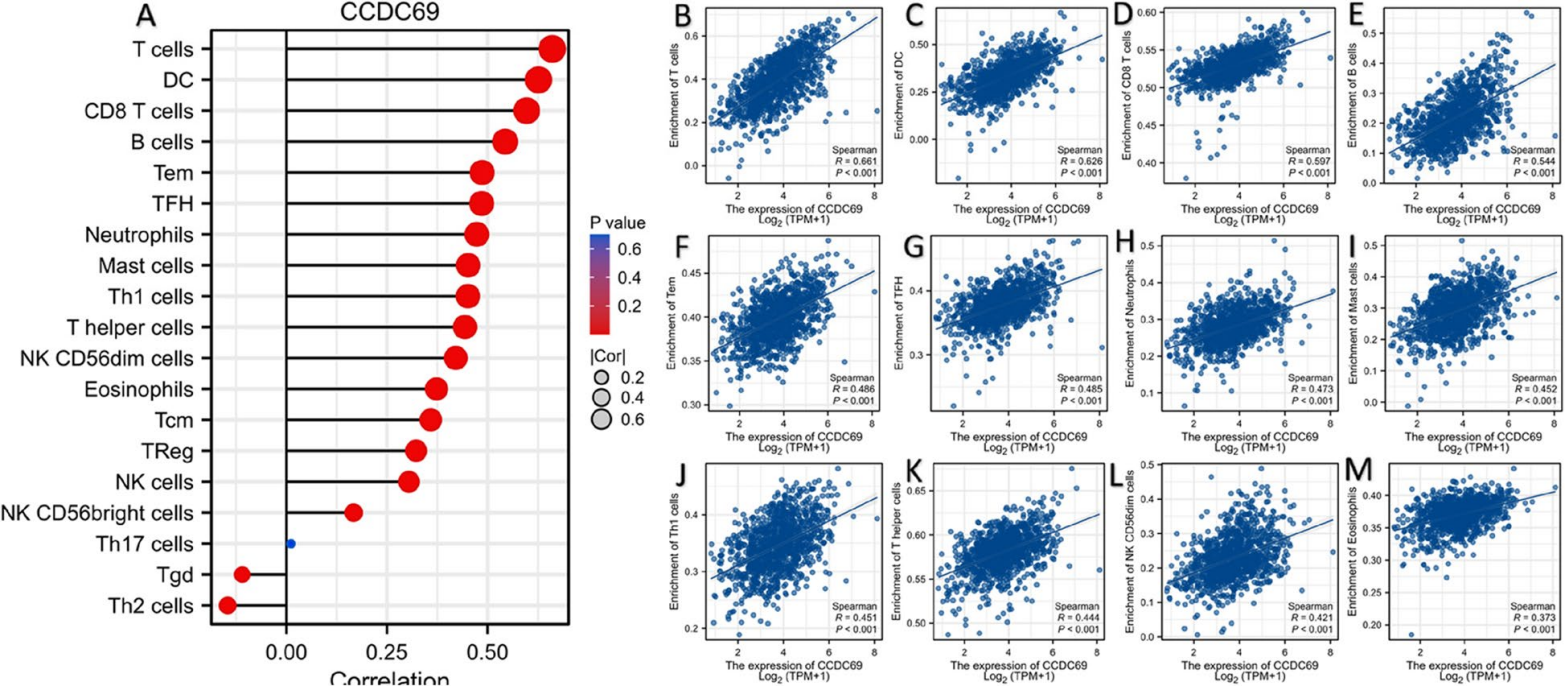
Correlation between CCDC69 with immune infiltration in breast cancer. **A** Correlation between CCDC69 expression and multiple kinds of tumor infiltrating immune cells in breast cancer generally. (B-M) Correlation between CCDC69 expression and (**B**) T cells, (**C**) DC cells, (**D**) CD8 + T cells, (**E**) B cells, (**F**) Tem cells, (**G**) TFH cells, (**H**) neutrophiles, (**I**) mast cells, (**J**) Th1 cells, (**K**) T helper cells, (**L**) NK CD56dim cells, and (**M**) eosinophils

We performed comprehensive analysis in TCGA database to analyze the correlations between CCDC69 expression and immune cells in breast cancer. Figure 8 A-M illustrated that CCDC69 expression was positively correlated with the infiltration level of T cells (especially CD8 + T cells), dendritic cells (DCs), B cells, T effector memory cells (Tems), T follicular helper cells (TFHs), neutrophiles, mast cells, type 1 T helper cells (Th1s), T helper cells, NK CD56dim cells, eosinophils, etc.

**Fig. 9:**
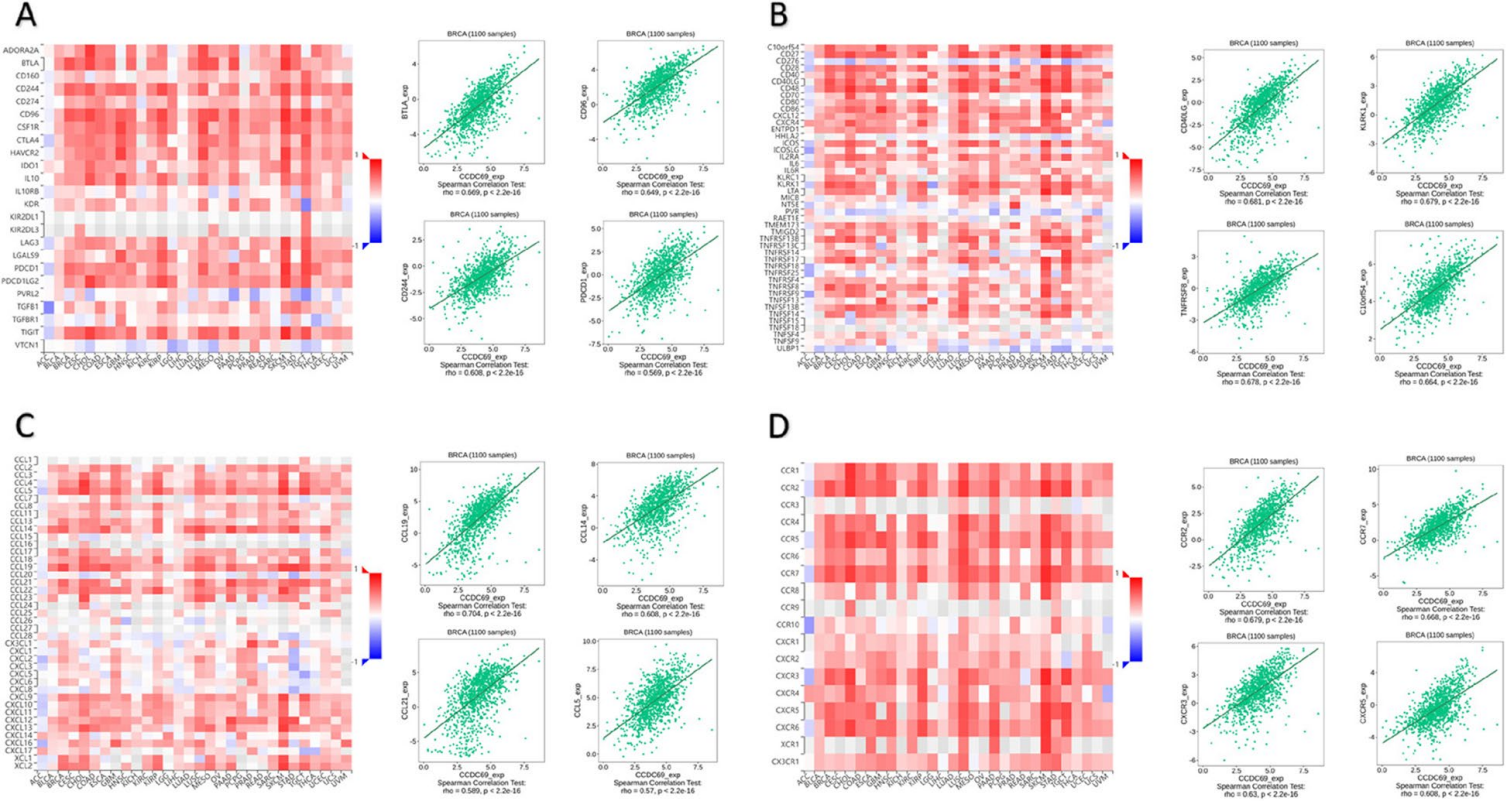
**A** Correlation between CD146 expression and immunoinhibitors, (**B**) immunostimulators, (**C**) chemokines, and (**D**) chemokine receptors in breast cancer available from the TISIDB database

The association of CCDC69 with immunomodulators and chemokines was further evaluated using the TISIDB database. Figure 9 A showed the strong correlations of CCDC69 with immunoinhibitors such as BTLA, CD96, CD244, and PDCD1. The expression of CCDC69 was also associated with immunostimulators, including CD40LG, KLRK1, TNFRSF8, and C10orf54 (Fig. 9B). Figure 9 C displayed that various chemokines, including CCL19, CCL14, CCL21, and CCL5, presented the greatest correlations with CCDC69 expression. Meanwhile, CCDC69 expression was significantly associated with chemokine receptors, including CCR2, CCR7, CXCR3, and CXCR5 (Fig. 9D). These outcomes revealed that CCDC69 functioned as an immunoregulatory factor in breast cancer.

### The correlation between CCDC69 and tumor immune microenvironment heterogeneity

**Fig. 10:**
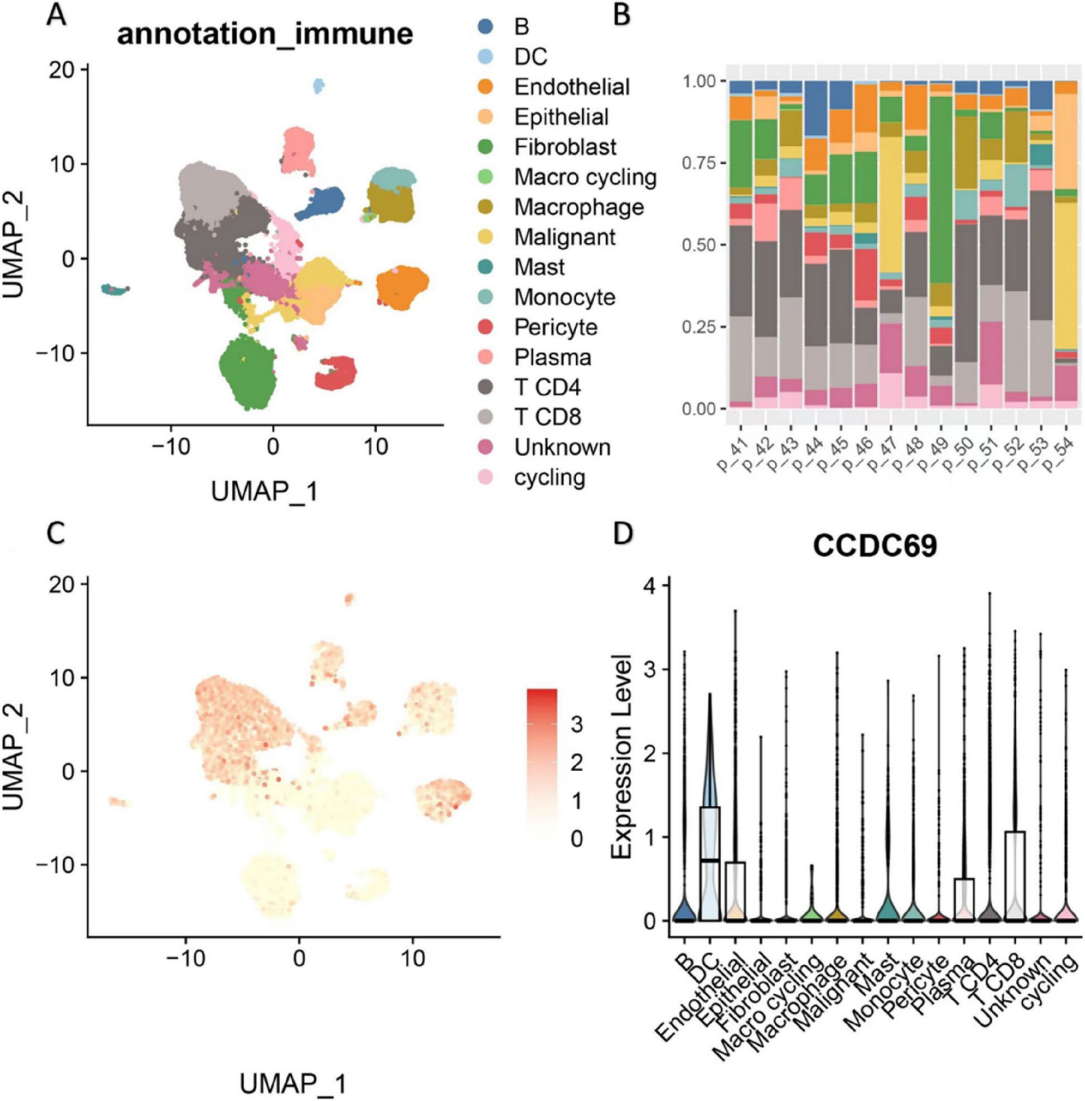
**A** UMAP plot of different cell distribution in BC_UNB_10X_E-MTAB-8107 dataset. **B** Different cell distribution level in different patients in BC_UNB_10X_E-MTAB-8107 dataset. **C** UMAP plot of CCDC69 expression in BC_UNB_10X_E-MTAB-8107 dataset. **D** CCDC69 expression in different cell types in BC_UNB_10X_E-MTAB-8107 dataset

**Fig. 11:**
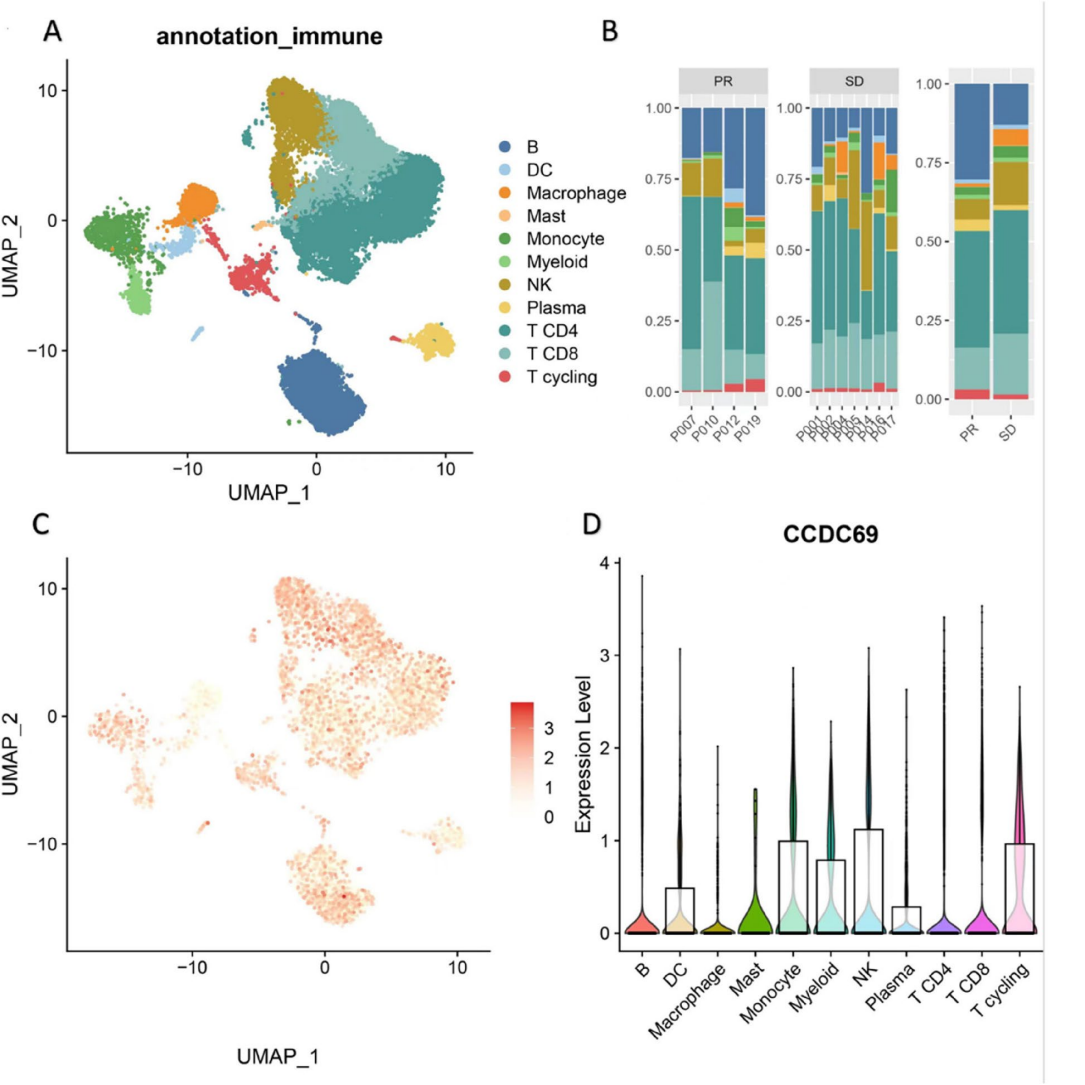
**A** UMAP plot of different cell distribution in TNBC_IMM_10X_GSE169246 dataset. **B** Different cell distribution level in different patients and response groups in TNBC_IMM_10X_GSE169246 dataset. **C** UMAP plot of CCDC69 expression in TNBC_IMM_10X_GSE169246 dataset. **D** CCDC69 expression in different cell types in TNBC_IMM_10X_GSE169246 dataset

In the BC_UNB_10X_E-MTAB-8107 dataset, a total of 15 types of cells (Fig. 10A) were observed, and the distribution of these cells in patients was as shown in Fig. 10B. It could be seen that CD4 + T cell and CD8 + T cell accounted for nearly half of cell distribution in a majority of the patients. Furthermore, we compared the expression of CCDC69 in different cell types in BC_UNB_10X_E-MTAB-8107 dataset, and different immune cell types such as DC, CD8 + T cell, B cell, and CD4 + T cell showed higher expression levels of CCDC69 (Fig. 10C, D) when compared with malignant and epithelial cells. In addition, we downloaded the immunotherapy dataset TNBC_IMM_10X_GSE169246 from the IMMUcan database containing 22 advanced TNBC patients, half of whom received atezolizumab (anti-PD-L1) plus paclitaxel. The other half received only paclitaxel, with the objective response rate (ORR) as the primary endpoint. Here, we evaluated CCDC69 expression in tumor immune microenvironment-associated immune cells using single-cell transcriptomes obtained from 11 tumors pretreated with atezolizumab plus paclitaxel. In the TNBC_IMM_10X_GSE169246 dataset, a total of 11 types of cells (Fig. 11A) were found, and the distribution of these cells in patients was as shown in Fig. 11B. It could be seen that CD4 + T cell and CD8 + T cell accounted for nearly half of cell distribution in a majority of the patients as well. We also compared the cell distribution difference between response and non-response patients after atezolizumab plus paclitaxel treatment. The proportion of B cells, CD4 + T cells, and CD8 + T cells in PR (response) patients was significantly higher than that in SD (non-response) patients. Furthermore, we compared the expression of CCDC69 in different cell types in the TNBC_IMM_10X_GSE169246 dataset, and different immune cell types such as NK, CD8 + T cells and B cells showed higher levels of CCDC69 expression (Fig. 11C-D).

#### CCDC69 expression indicates immunotherapy response

**Fig. 12:**
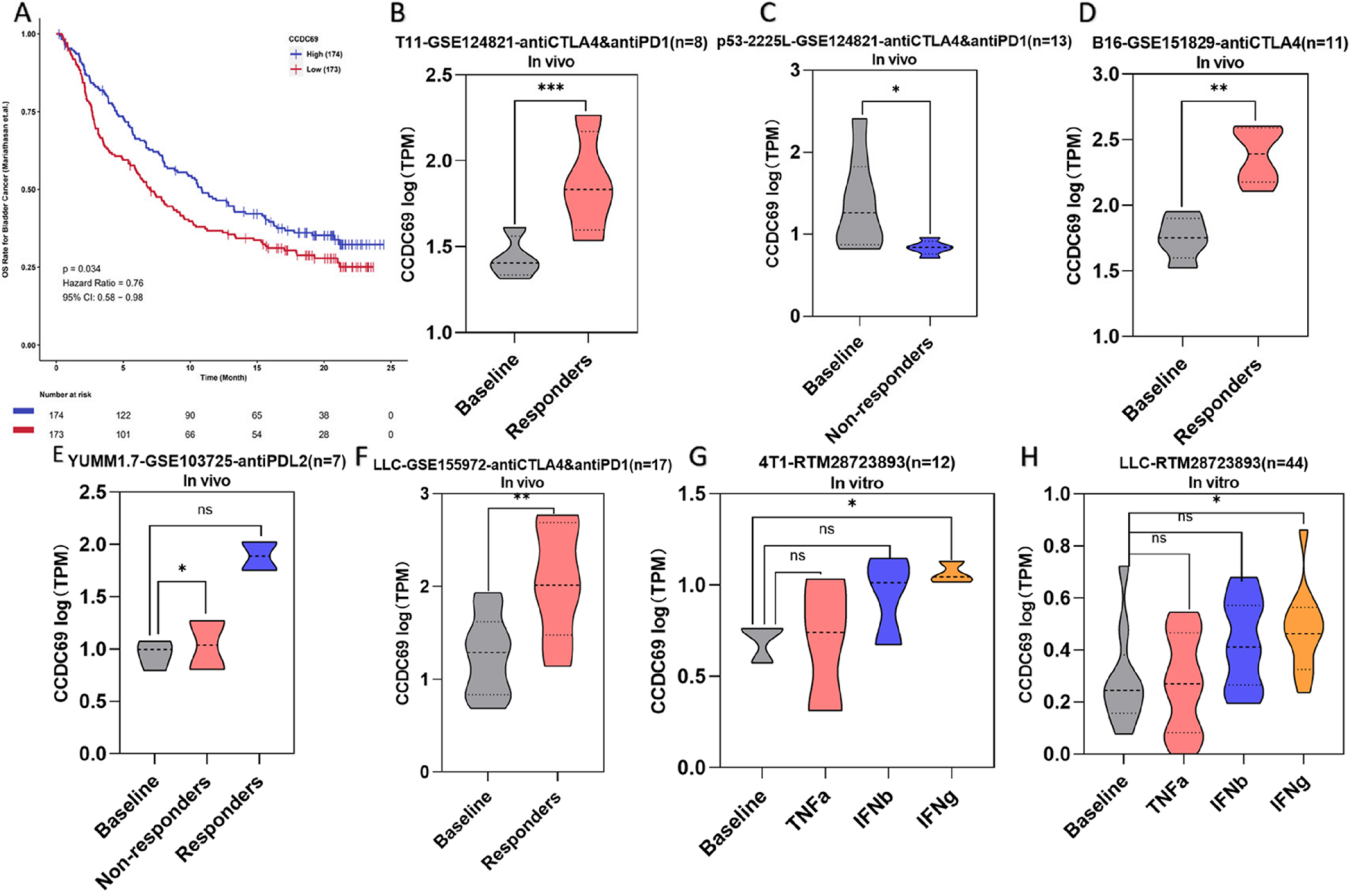
**A** The KM survival curve of bladder cancer patients in CCDC69 high expression group and low expression group. **B-F** CCDC69 expression in tumor tissues with different ICI response status in (**B**) T11 in vivo model, (**C**) p53-2225 L in vivo model, (**D**) B16 in vivo model, (**E**) YUMM1.7 in vivo model, and (**F**) LLC in vivo model. (**G-H**) CCDC69 expression after cytokines treatment in (**G**) 4T1 in vitro model and (**H**) LLC in vitro model. ***p < 0.001, **p < 0.01, *p < 0.05, ns: not significant

In the ICI-treated patient cohort of Camoip database, we found that higher expression level of CCDC69 could predict better immunotherapy benefits in bladder cancer, as shown by OS (HR = 0.76, 95%CI (0.58–0.98), *p* = 0.034) (Fig. 12A). We further evaluated the prognosis value of CCDC69 in immunotherapy in mouse tissues using TISMO database. Higher CCDC69 expression in the responder groups and lower CCDC69 expression in the non-responder groups after ICI treatments were observed in the breast cancer model (T11, p53-2225 L) (Fig. 12B, C), melanoma model (B16, YUMM1.7) (Fig. 12D, E), and lung cancer model (LCC) in vivo (Fig. 12F). We also found that CCDC69 was significantly upregulated after exposure to interferon (IFN)-gamma in 4T1 cells (breast cancer) (Fig. 12G) and LLC cells (lung cancer) (Fig. 12H) in vitro.

#### CCDC69 expression is correlated with immune-related scores in breast cancer

**Fig. 13:**
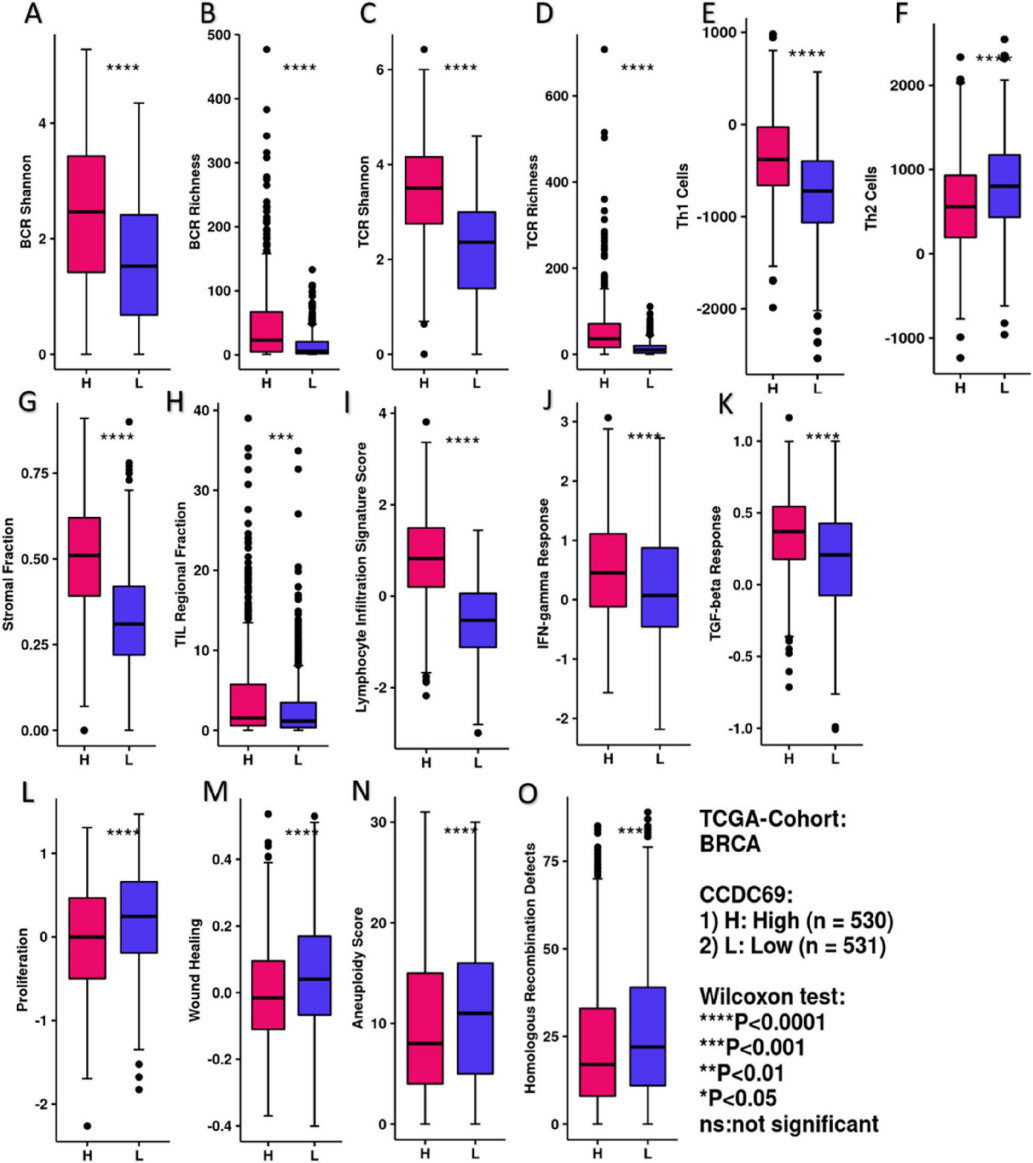
**A** BCR shannon, (**B**) BCR richness, (**C**) TCR shannon, (**D**) TCR richness, (**E**) Th1 cells, (**F**) Th2 cells, (**G**) stromal fraction, (**H**) TIL regional fraction, (**I**) lymphocyte infiltration signature score, (**J**) IFN-gamma response, (**K**) TGF-beta response, (**L**) proliferation, (**M**) wound healing, (**N**) aneuploidy score, and (**O**) homologous recombination defects comparison between CCDC69 high expression group and CCDC69 low expression group. ***p < 0.001, **p < 0.01, *p < 0.05, ns: not significant

The correlations between CCDC69 expression and immune infiltrating cells were shown in Fig. 13A-F. CCDC69 was positively correlated with the Shannon and richness of B cell receptor (BCR), T cell receptor (TCR), and Th1, Th2 cells. CCDC69 was demonstrated to be positively correlated with stromal fraction (Fig. 13G), tumor infiltrating lymphocyte (TIL) fraction (Fig. 13H), and lymphocyte infiltration signature score (Fig. 13I). Moreover, high expression of CCDC69 predicted better IFN-gamma and TGF-beta response (Fig. 13J-K). Some malignant signs such as proliferation, wound healing, aneuploidy score, and homologous recombination defects were negatively correlated with CCDC69 expression (Fig. 13L-O).

#### CCDC69 expression predicts the response of multiple chemotherapeutic strategies

**Fig. 14:**
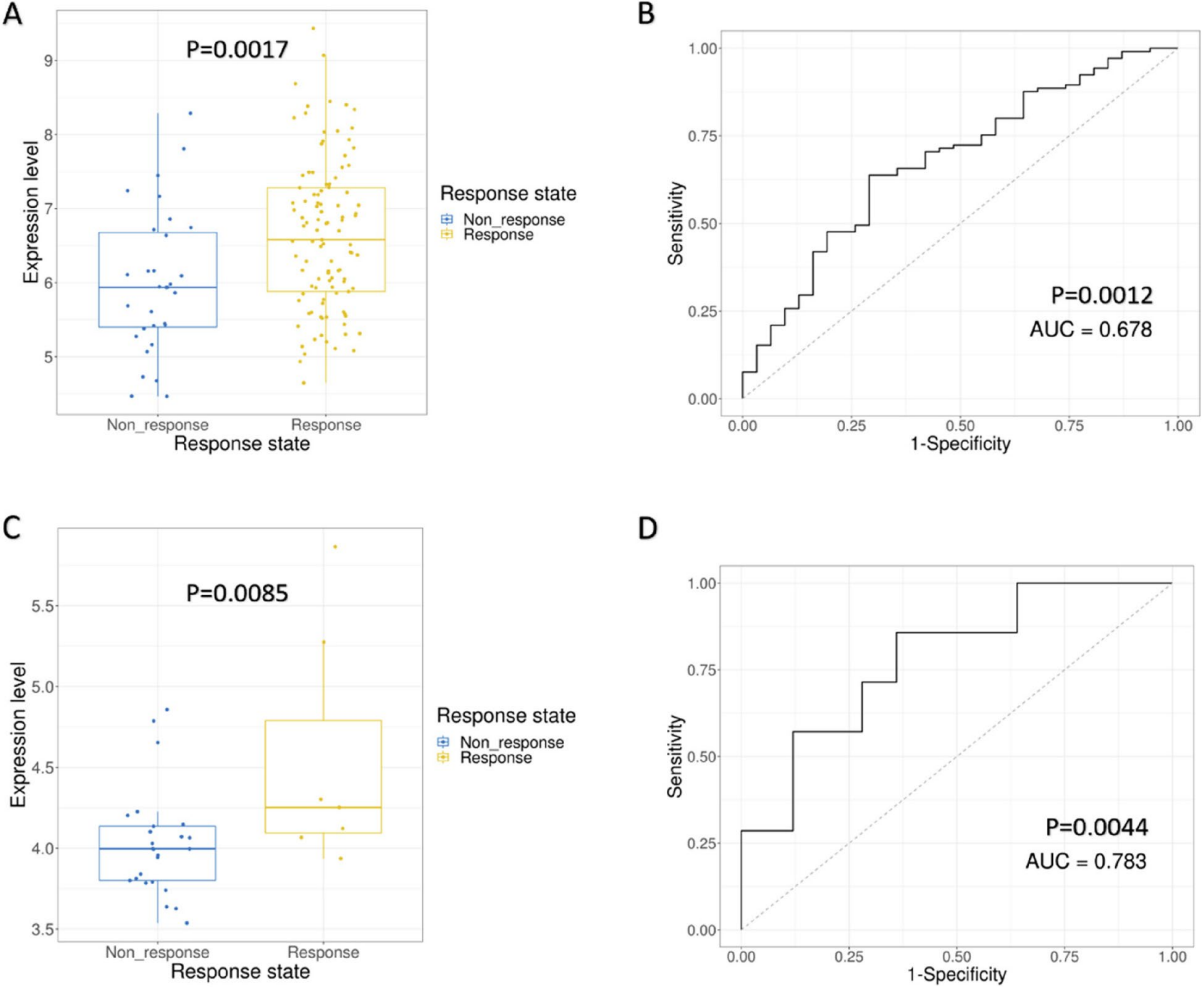
**A** CCDC69 expression in breast cancer tissues with different CDI response status. **B** The receiver operator characteristic (ROC) curve of CCDC69 in predicting the response status of CDI. **C** CCDC69 expression in breast cancer tissues with different CEFCD response status. **D** The receiver operator characteristic (ROC) curve of CCDC69 in predicting the response status of CEFCD.

As shown in Fig. 14, high expression of CCDC69 was observed in both Cyclophosphamide + Doxorubicin + Ixabepilone (CDI) treatment response group (Fig. 14A, *p* = 0.0017) and Cyclophosphamide + Epirubicin + Fluorouracil + Capecitabine + Docetaxel (CEFCD) treatment response group (Fig. 14C, *p* = 0.0085). Moreover, CCDC69 was a reliable predictor for the response of CDI treatment (Fig. 14B) and CEFCD treatment (Fig. 14D) with the area under the curve (AUC) of 0.678 and 0.783, repsectively.

## Discussion

Breast cancer is clinically divided into four molecular subtypes, namely, luminal A and B; HER2-positive, and triple-negative breast cancer (TNBC) by the expression of ER, PR, HER2, and KI-67, but such a classification cannot fully realize personalized precision medicine for treating breast cancer. More targets and biomarkers and more precise molecular subtyping should be explored to improve therapeutic efficacy and reduce adverse side effects. With the continuous development of sequencing platforms, in-depth bioinformatics analysis based on genomic data has been increasingly applied for biomarker prediction, prognosis analysis, and targeted therapy in cancers as well as some other diseases [29–32]. In this study, we conducted a series of bioinformatics analyses on the basis of multiple bioinformatics databases and further verified the results in clinical samples. We found that CCDC69 was a downregulated gene in breast cancer tissues compared with normal tissues, and demonstrated the prognosis value of CCDC69 and its protective effects on breast cancer from multiple aspects. CCDC69 is expected to be an effective biomarker to predict the survival of breast cancer patients, facilitating the early diagnosis based on molecular subtypes, histological subtypes as well as lymph nodes metastasis of breast cancer. Besides, the expression of CCDC69 is also a useful predictor of immunotherapy response in multiple cancers. Therefore, personalize treatment and management strategies can be developed appropriately based on the combination of CCDC69 expression level and other factors.

It is known that tumor immune infiltration could affect the sensitivity to chemotherapy, radiotherapy, immunotherapy and also the survival of cancer patients [33–35]. In our research, we detected strong correlations between CCDC69 expression and multiple immune cells infiltration. The favorable effects of T cells including CD8 + T cells [36, 37] and some subtypes of CD4 + T cells such as TFH [38] and Th1 [38] in breast cancer have been revealed. DCs act as a tumor antigen transporter to initiate T cell activation, which is required for T cell-dependent immunity and response to ICI therapy [39, 40]. Moreover, the anti-tumor effects of B cells [41, 42], eosinophils [43], and NK CD56dim cells [44] in breast cancer have been proven. However, the biological functions of neutrophils [45, 46] and mast cells [47] in breast cancer are still controversial. The specific roles of CCDC69 in the biological processes of neutrophils and mast cells in breast cancer are still under exploration, and future study could analyze the function of the both cells in breast cancer. These results revealed that high expression of CCDC69 indicated favorable prognosis in breast cancer possibly through promoting T cells proliferation and activation and anti-tumor immunity.

Due to the emerging role of immune system in breast cancer progression and prognosis, immunotherapy, especially ICIs, has become a hot research subject [48]. The antibodies of programmed cell death receptor 1 (PD-1), programmed cell death 1 ligand 1 (PD-L1), and cytotoxic T-lymphocyte-associated antigen-4 (CTLA-4) have been applied as ICIs for the treatment of breast cancer. The immunotherapy response in breast cancer is associated with T cell infiltration [48], while higher T cell infiltration level predicts better ICIs treatment response [49]. In our results, higher CCDC69 expression suggested more ICIs treatment benefits, and we speculated that CCDC69 can improve immunotherapy efficiency by promoting the activation of T cells. Moreover, IFN, a kind of cytokine, has been applied in the immunotherapy of cancers [50]. In breast cancer, the major source of IFN-gamma is Th1 cells and CD8 + T cells [51]. The production of IFN-gamma can boost anti-tumoral T cell response [52, 53]. We observed that the application of IFN-gamma upregulated the expression of CCDC69 in vitro in our results, and CCDC69 possibly participated in the regulatory process when IFN-gamma activating T cell responses.

Currently, ICIs targeting PD-L1 has been widely used as an effective therapeutic option for treating TNBC patients [54]. However, the clinical practice of ICIs in the therapy of ER/PR + breast cancer patients was not satisfactory [55]. Our results indicated that the CCDC69 was downregulated in ER/PR + breast cancer samples, while the upregulation of CCDC69 was correlated with high level of TILs, especially T cells, in breast cancer. Accumulating evidence has shown a favorable value of TILs in the prognosis of TNBC and HER + breast cancer patients, but the role of TILs in luminal breast cancer was still unclear [56]. A deeper understanding of CCDC69 and its effects on the regulating of immune infiltration could help improve the therapeutic effect of ICIs on luminal breast cancer.

In a word, CCDC69 was downregulated in breast cancer, and it was correlated with a better clinical prognosis. Our results demonstrated that CCDC69 regulated multiple immunity-related mechanisms and affected the immune cell infiltration, especially T cells and DC cells, in breast cancer. Moreover, CCDC69 played important roles in the immunotherapy responses and higher expression level predicted better immunotherapy responses. Further researches could be conducted to explore the exact mechanisms of CCDC69 in breast cancer immune microenvironment regulation and immunotherapy response.

### Supplementary Information

**Additional file 1: Supplementary table. 1.** Univariate and multivariate cox analysis of the relationship between CCDC69 expression and DSS of TCGA breast cancer patients. **Supplementary table. 2.** Univariate and multivariate cox analysis of the relationship between CCDC69 expression and PFI of TCGA breast cancer patients. **Supplementary figure. 1.** The p-values and log2FC values of CCDCC69 differential expression in pan-cancer tissues compared with adjacent normal tissues in GENT2 database. **Supplementary figure. 2.** Forest map of multivariate cox analysis of the relationship between CCDC69 expression and DSS of TCGA breast cancer patients. **Supplementary figure. 3.** Forest map of multivariate cox analysis of the relationship between CCDC69 expression and PFI of TCGA breast cancer patients.

## Data Availability

The datasets used and/or analyzed during the current study available from the corresponding author on reasonable request.

## Abbreviations

CCDC69: Coiled-coil domain-containing protein 69
PPI: Protein-protein interaction
GSEA: Gene set enrichment analysis
qRT-PCR: Quantitative reverse transcriptase-polymerase chain reaction
IHC: Immunohistochemical
GENT2: Gene Expression patterns across Normal and Tumor tissues database
TCGA: The cancer genome altas
TPM: Transcripts per million reads
KM: Kaplan-meier
DEGs: Differentially expressed genes
bc-GenExMiner: Breast cancer Gene-Expression miner
TISIDB: Tumor-Immune System Interactions database
ICI: Immune checkpoint inhibitor
TISMO: Tumor Immune Syngeneic Mouse database
ctr-db: Cancer Treatment Response gene signature database
OS: Overall survival
DSS: Disease-specific survival
PFI: Progression-free interval
RFS: Recurrence-free survival
DMFS: Distant metastasis-free survival
HR: Hazard ratio
CI: Confidence interval
NK: Natural killing
ER: Estrogen receptor
PR: Progesterone receptor
HER2: Human epidermal growth factor receptor 2
DC: Dendritic cell
TFH: T follicular helper cell
Tem: T effector memory cell
Th1: Type 1 T helper cell
Th17: Type 17 T helper cell
Th2: Type 2 T helper cell
Treg: Regulatory T cell
Tcm: T central memory cell
Tgd: T gamma delta cell
ORR: Objective response rate
IFN: Interferon
BCR: B cell receptor
TCR: T cell receptor
TIL: Tumor infiltration lymphocyte
CDI: Cyclophosphamide +doxorubicin +ixabepilone
CEFCD: Cyclophosphamide +epirubicin +fluorouracil +capecitabine +docetaxel
AUC: Area under the curve
ROC: Receiver operator characteristic
TNBC: Triple-negative breast cancer
PD-1: Programmed cell death receptor 1
PD-L1: Programmed cell death 1 ligand 1
CTLA-4: Cytotoxic T-lymphocyte-associated antigen-4
