# Supplementary material for: High expression of CCDC69 is correlated with immunotherapy response and protective effects on breast cancer
Source: BMC Cancer. 2023 Oct 12;23:974. doi: 10.1186/s12885-023-11411-2 (PMC10571395; doi:10.1186/s12885-023-11411-2)

Supplementary table. 1: Univariate and multivariate cox analysis of the relationship between CCDC69 expression and DSS of TCGA breast cancer patients.

| Characteristics | Total(N) | Univariate analysis | |  | Multivariate analysis | |
| --- | --- | --- | --- | --- | --- | --- |
|  |  | Hazard ratio (95% CI) | P value |  | Hazard ratio (95% CI) | P value |
| CCDC69 | 1062 |  |  |  |  |  |
| Low | 531 | Reference |  |  |  |  |
| High | 531 | 0.540 (0.346-0.841) | **0.006** |  | 0.580 (0.345-0.977) | **0.041** |
| Age | 1062 |  |  |  |  |  |
| <=60 | 590 | Reference |  |  |  |  |
| >60 | 472 | 1.445 (0.941-2.219) | 0.093 |  | 1.644 (0.993-2.722) | 0.053 |
| Race | 974 |  |  |  |  |  |
| Asian | 60 | Reference |  |  |  |  |
| Black or African American | 180 | 0.913 (0.266-3.130) | 0.884 |  |  |  |
| White | 734 | 0.778 (0.243-2.493) | 0.673 |  |  |  |
| T stage | 1059 |  |  |  |  |  |
| T1 | 274 | Reference |  |  |  |  |
| T2 | 618 | 1.551 (0.877-2.741) | 0.131 |  | 1.206 (0.618-2.356) | 0.583 |
| T3 | 133 | 1.769 (0.849-3.686) | 0.128 |  | 1.140 (0.444-2.925) | 0.785 |
| T4 | 34 | 6.700 (3.000-14.965) | **<0.001** |  | 2.979 (1.109-8.006) | **0.030** |
| N stage | 1044 |  |  |  |  |  |
| N0 | 511 | Reference |  |  |  |  |
| N1 | 347 | 3.390 (1.926-5.967) | **<0.001** |  | 2.944 (1.590-5.450) | **<0.001** |
| N2 | 112 | 3.752 (1.756-8.020) | **<0.001** |  | 4.620 (2.065-10.333) | **<0.001** |
| N3 | 74 | 7.123 (3.317-15.296) | **<0.001** |  | 4.978 (1.705-14.532) | **0.003** |
| M stage | 903 |  |  |  |  |  |
| M0 | 884 | Reference |  |  |  |  |
| M1 | 19 | 7.454 (3.988-13.931) | **<0.001** |  | 1.903 (0.817-4.432) | 0.136 |
| PAM50 | 1023 |  |  |  |  |  |
| LumA | 554 | Reference |  |  |  |  |
| LumB | 199 | 1.685 (0.940-3.021) | 0.080 |  | 1.109 (0.575-2.142) | 0.757 |
| Her2 | 80 | 2.780 (1.407-5.495) | **0.003** |  | 2.352 (1.081-5.117) | **0.031** |
| Basal | 190 | 1.813 (1.051-3.127) | **0.032** |  | 2.274 (1.227-4.212) | **0.009** |
| radiation_therapy | 977 |  |  |  |  |  |
| No | 430 | Reference |  |  |  |  |
| Yes | 547 | 0.791 (0.483-1.295) | 0.351 |  |  |  |

Supplementary table. 2: Univariate and multivariate cox analysis of the relationship between CCDC69 expression and PFI of TCGA breast cancer patients.

| Characteristics | Total(N) | Univariate analysis | |  | Multivariate analysis | |
| --- | --- | --- | --- | --- | --- | --- |
|  |  | Hazard ratio (95% CI) | P value |  | Hazard ratio (95% CI) | P value |
| CCDC69 | 1082 |  |  |  |  |  |
| Low | 541 | Reference |  |  |  |  |
| High | 541 | 0.643 (0.463-0.894) | **0.009** |  | 0.572 (0.384-0.852) | **0.006** |
| Age | 1082 |  |  |  |  |  |
| <=60 | 601 | Reference |  |  |  |  |
| >60 | 481 | 1.253 (0.904-1.738) | 0.175 |  |  |  |
| Race | 993 |  |  |  |  |  |
| Asian | 60 | Reference |  |  |  |  |
| Black or African American | 180 | 0.947 (0.364-2.465) | 0.912 |  |  |  |
| White | 753 | 0.832 (0.338-2.049) | 0.689 |  |  |  |
| T stage | 1079 |  |  |  |  |  |
| T1 | 276 | Reference |  |  |  |  |
| T2 | 629 | 1.615 (1.042-2.501) | **0.032** |  | 1.371 (0.819-2.297) | 0.230 |
| T3 | 139 | 2.213 (1.290-3.798) | **0.004** |  | 1.374 (0.679-2.784) | 0.377 |
| T4 | 35 | 6.258 (3.262-12.008) | **<0.001** |  | 2.447 (1.006-5.953) | **0.048** |
| N stage | 1063 |  |  |  |  |  |
| N0 | 514 | Reference |  |  |  |  |
| N1 | 357 | 1.981 (1.331-2.948) | **<0.001** |  | 1.769 (1.133-2.760) | **0.012** |
| N2 | 116 | 2.481 (1.441-4.272) | **0.001** |  | 2.629 (1.454-4.754) | **0.001** |
| N3 | 76 | 4.961 (2.833-8.688) | **<0.001** |  | 3.196 (1.365-7.482) | **0.007** |
| M stage | 922 |  |  |  |  |  |
| M0 | 902 | Reference |  |  |  |  |
| M1 | 20 | 8.315 (4.829-14.315) | **<0.001** |  | 3.125 (1.461-6.682) | **0.003** |
| PAM50 | 1042 |  |  |  |  |  |
| LumA | 561 | Reference |  |  |  |  |
| LumB | 204 | 1.209 (0.762-1.918) | 0.420 |  | 0.873 (0.515-1.480) | 0.614 |
| Her2 | 82 | 2.129 (1.231-3.685) | **0.007** |  | 1.742 (0.939-3.232) | 0.079 |
| Basal | 195 | 1.491 (0.981-2.265) | 0.061 |  | 1.892 (1.187-3.017) | **0.007** |
| radiation_therapy | 986 |  |  |  |  |  |
| No | 434 | Reference |  |  |  |  |
| Yes | 552 | 0.899 (0.631-1.281) | 0.555 |  |  |  |

Supplementary figure. 1: The p-values and log2FC values of CCDCC69 differential expression in pan-cancer tissues compared with adjacent normal tissues in GENT2 database.


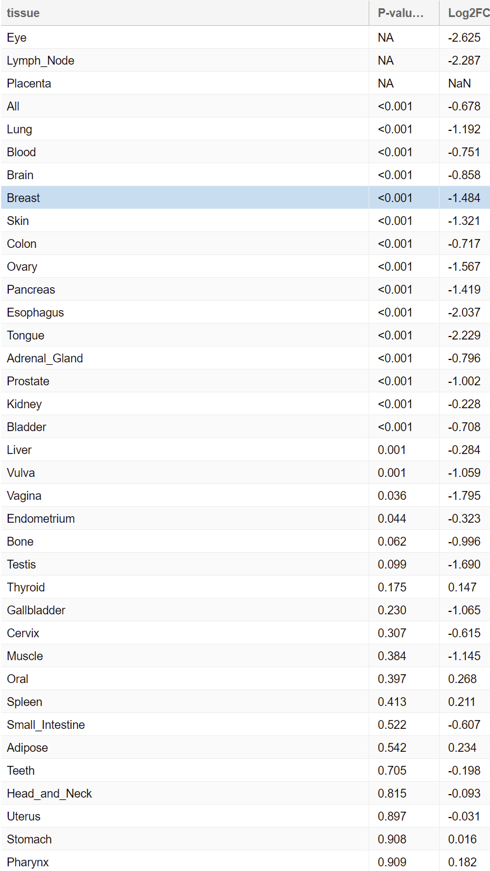


Supplementary figure. 2: Forest map of multivariate cox analysis of the relationship between CCDC69 expression and DSS of TCGA breast cancer patients.


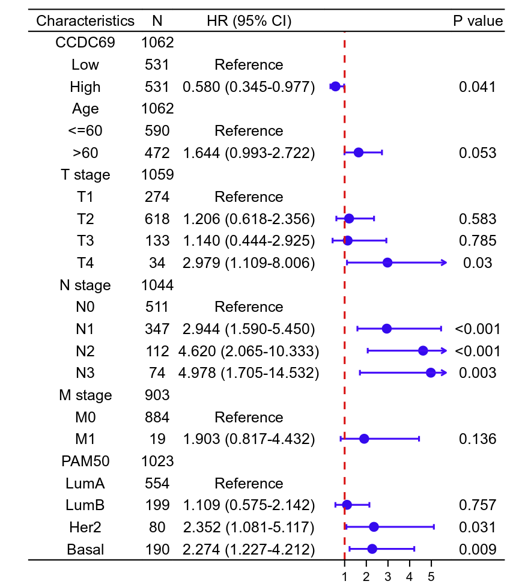


Supplementary figure. 3：Forest map of multivariate cox analysis of the relationship between CCDC69 expression and PFI of TCGA breast cancer patients.


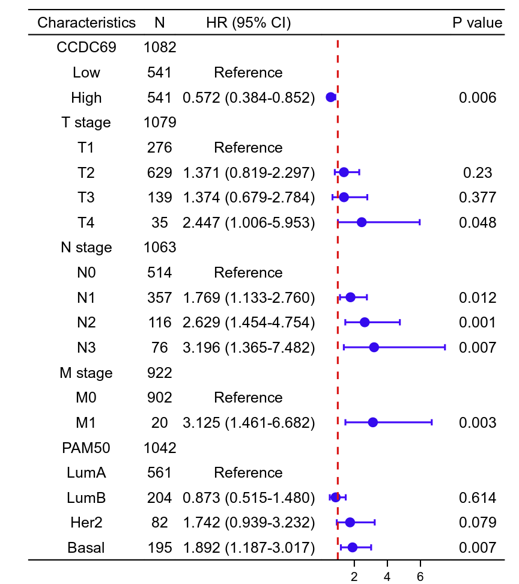

Supplement: Supplementary file 1 — Additional file 1: Supplementary table. 1. Univariate and multivariate cox analysis of the relationship between CCDC69 expression and DSS of TCGA breast cancer patients. Supplementary table. 2. Univariate and multivariate cox analysis of the relationship between CCDC69 expression and PFI of TCGA breast cancer patients. Supplementary figure. 1. The p-values and log2FC values of CCDCC69 differential expression in pan-cancer tissues compared with adjacent normal tissues in GENT2 database. Supplementary figure. 2. Forest map of multivariate cox analysis of the relationship between CCDC69 expression and DSS of TCGA breast cancer patients. Supplementary figure. 3. Forest map of multivariate cox analysis of the relationship between CCDC69 expression and PFI of TCGA breast cancer patients. [file 12885_2023_11411_MOESM1_ESM.docx]
